# Supplementary material for: Uptake Quantification of Antigen Carried by Nanoparticles and Its Impact on Carrier Adjuvanticity Evaluation
Source: Vaccines (Basel). 2023 Dec 26;12(1):28. doi: 10.3390/vaccines12010028 (PMC10818693; doi:10.3390/vaccines12010028)
Supplement: Supplementary file 1 [file vaccines-12-00028-s001.zip › vaccines-2662458-supplementary.pdf]

Supporting Materials

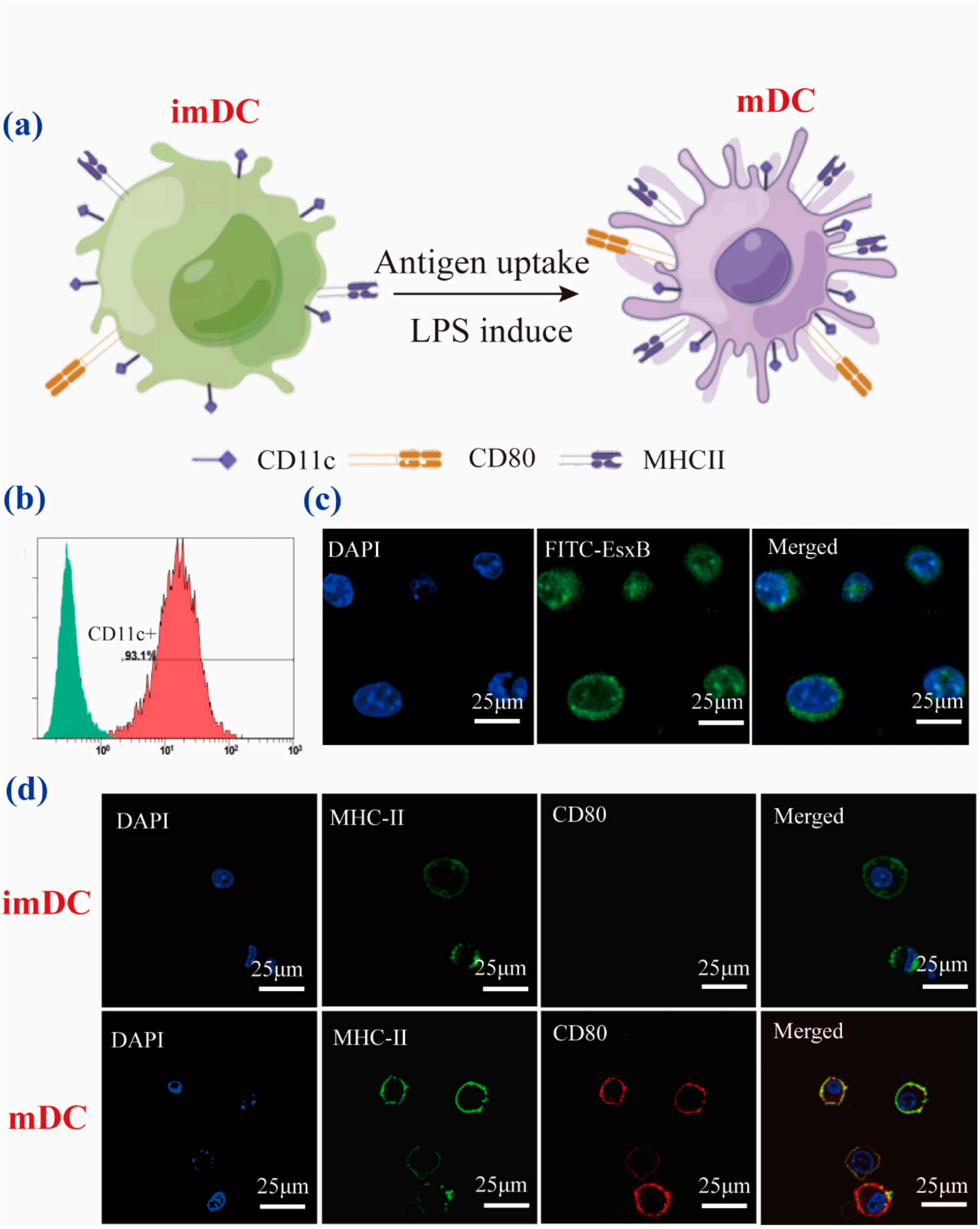

Fig.S1. BMDC characterization. (a). Schematic of BMDC maturation process after antigen uptake or LPS induction, especially the changes in the number of molecules on the cell surface. (b). Flow cytometry analysis of CD11c$^+$ expression

on BMDCs. The CD11c^{+} molecule was stained by PE-CD11c. (c). Confocal microscopy images of BMDCs after endocytosis of FITC-EsxB. Nuclear was stained with DAPI (Ex/Em 360/460 nm), and EsxB was labeled by FITC (Ex/Em 488/525 nm). (d). MHC Ⅱ and CD80 expressions on cell surface was determined by confocal microscopy. Immature DCs were induced by LPS (1μg/mL) for 12 h to obtain mature DCs. MHCII was stained by Alexa flour@488-MHCII (Ex/Em 488/520 nm) and CD80 was stained by Alexa flour@594-CD80 (Ex/Em 590/617 nm).

We exacted bone marrow cells from mice and cultured with GM-CSF to obtain BMDC. The flow cytometry analysis of cells showed that the CD11c^{+} positive cells accounted for 93.1% which indicated the BMDC purity is relatively high. Besides, the endocytosis results of EsxB showed the antigen uptaken ability of BMDC. The two states of DCs were detected by confocal microscopy, immature DCs have the high ability on antigen uptake, the low ability on antigen presentation, while the mature DCs stimulated by LPS showed conversely ability, Meanwhile, the surface molecules such as MHCII and CD80 expression higher in the mDCs group.

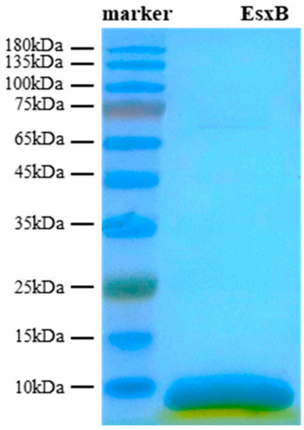

Fig. S2. Expression and purification of EsxB antigen. EsxB antigen was analyzed by SDS-PAGE, Lane 1, Marker, Lane 2, 6×His-tag rEsxB. Expected molecular weight of rEsxB is ~11.5 kDa (104 aa).

The purified EsxB protein was examined, and SDS-PAGE results showed a specific band at 11.5kDa, being consistent with the molecular weight of EsxB.

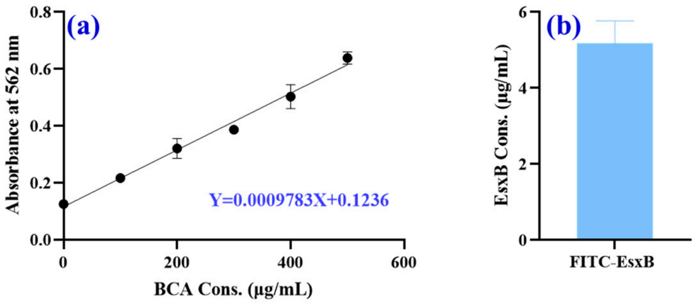

Fig.S3. Analysis of FITC-EsxB amount using the standard BCA method[1]: (a) The standard curve for determining the concentration of protein by BCA assay, (Absorbance was measured at 562 nm, R^2=0.998). (n=3). (b) Concentration determination for FITC-EsxB sample by BCA assay according to the standard curve shown in part (a).

We analyzed the protein concentration of FITC-EsxB by BCA method, and

the calculated concentration is 5.17 μg/mL.

Table.S1. The size distribution, zeta potential, Polydispersity index (PdI), encapsulation efficiency (EE) and loading capacity (LC) of FITC-EsxB-PLGA NPs. The data are presented as means ± SD (n = 6, analyzed by one-way ANOVA.). P value < 0.05 was considered as statistically significant (* $p < 0.05$; ** $p < 0.01$; *** $p < 0.001$; **** $p < 0.0001$). No significance was found in the data comparison.

Formulations	Size (nm)	ζ (mV)	PDI	EE (%)	LC(%)
FITC-EsxB- PLGA NPs	179.4±9.37	-22.2±2.6	0.232±0.009	30.7±9.02	3.3±0.06

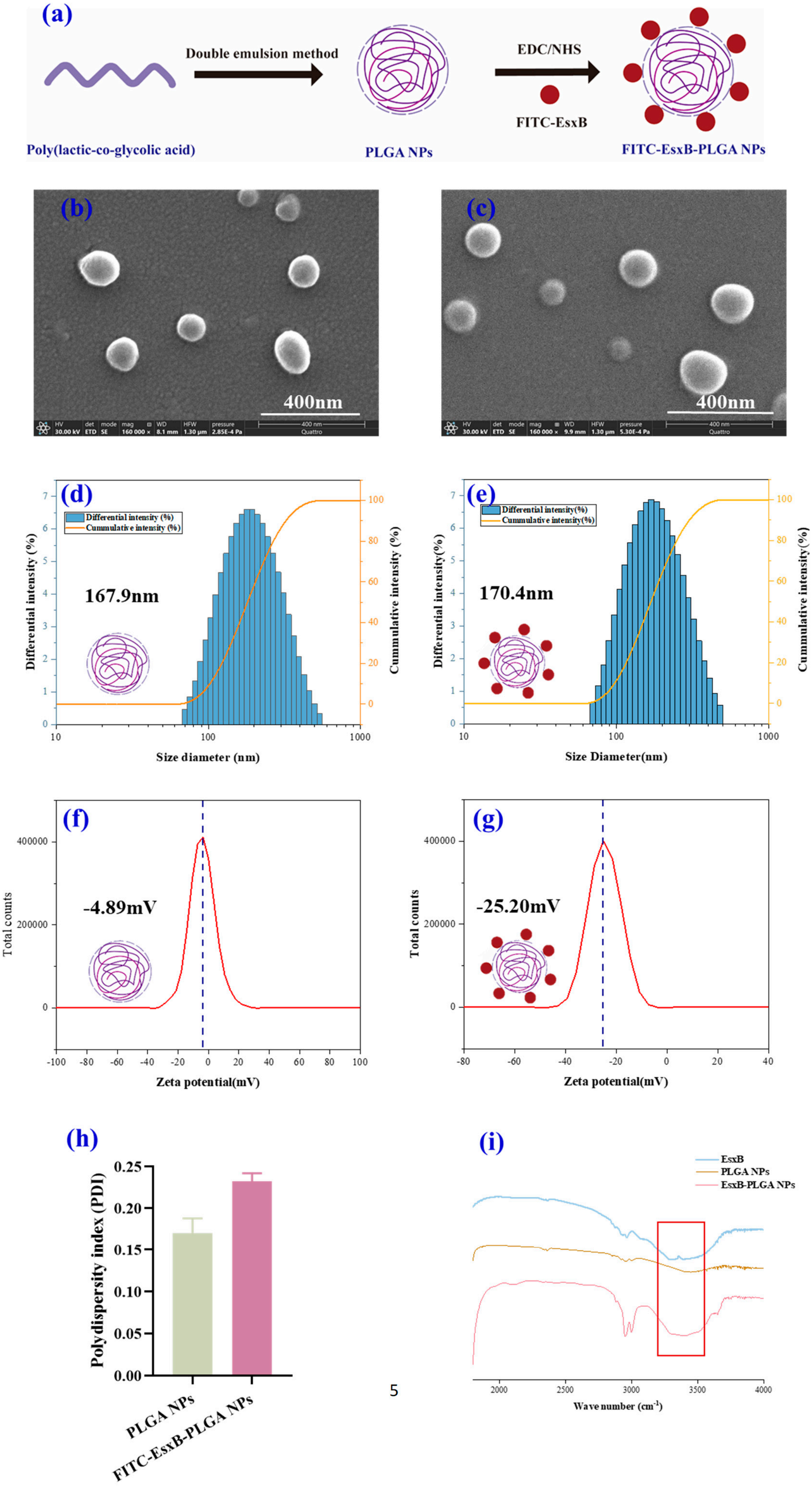
(a)
Double emulsion method
EDC/NHS
FITC-EsxB
Poly(lactic-co-glycolic acid)
PLGA NPs
FITC-EsxB-PLGA NPs
(b)
400nm
(c)
400nm
(d)
Differential intensity (%)
Cummulative intensity (%)
167.9nm
Size diameter (nm)
(e)
Differential intensity(%)
Cummulative intensity(%)
170.4nm
Size Diameter(nm)
(f)
-4.89mV
Total counts
Zeta potential(mV)
(g)
-25.20mV
Total counts
Zeta potential(mV)
(h)
Polydispersity index (PDI)
PLGA NPs
FITC-EsxB-PLGA NPs
(i)
EsxB
PLGA NPs
EsxB-PLGA NPs
Wave number (cm-1)

Fig.S4. Preparation and characterization of the FITC-EsxB-PLGA NPs. (a). Schematic of FITC-EsxB-PLGA NPs synthesis. SEM analysis of (b) PLGA NPs and (c) FITC-EsxB-PLGA NPs. Hydrodynamic diameter of (d) PLGA NPs and (e) FITC-EsxB-PLGA NPs was measured by DLS. Zeta potential of (f) PLGA NPs and (g) FITC-EsxB-PLGA NPs was determined by DLS. (h) . Polydispersity index (PDI) of PLGA NPs and FITC-EsxB-PLGA NPs was also determined by DLS (i) FTIR spectra was taken from EsxB, PLGA NPs, and the EsxB-PLGA NPs samples.

PLGA is a biocompatible material that had been approved by the FDA for medical device application[2]. PLGA nanoparticles as the antigen carriers have excellent capacities for sustained release of antigen over a prolonged time, long systemic circulation half-lives, and the potential for targeting the APCs to achieve the higher maturation and antigen presentation of APC[3].

The NP vaccines were prepared by a W/O/W double-emulsion solvent evaporation method **(Fig. S4a)**. It revealed advantages such as being a simple process and exhibiting customized efficacy. The various NP vaccine formulations and their physical properties are listed in **Table S1**. The NP vaccines showed a mean particle size of close to 180 nm, a monodispersed population (PdI=0.232), a negative surface charge, EEs about 30.7%, and LCs about 3.3%. In addition, the spherical morphologies of PLGA NPs and FITC-EsxB-PLGA NPs dispersed as individual particles were observed in SEM

graphs (**Fig. S4b and 4c**). The uniform size distributions evaluated by SEM were consistent with results obtained from DLS (**Fig. S4d and 4e**).

In the FTIR spectra taken from samples of EsxB, PLGA NPs, and their corresponding EsxB conjugates (**Fig. S4g.**), the bimodal due to the N-H stretching vibration in $-NH_2$ in the pure EsxB (~3500 cm^{-1}) disappeared in EsxB-PLGA NPs samples. Instead, the stretching vibration of -NH- (characteristic of the amide bond) can be found[4], suggesting the formation of amide bond, and thus successful binding of EsxB to the NPs.

The EE% is the ratio of the binding amount to the input amount of EsxB protein. In order to get more EsxB conjugated onto the PLGA nanoparticle surface, we increase the input amount of EsxB, leading to low EE%. Since the molecular weight of EsxB is about 11.5 kDa, the steric hindrance is large which lead to relative low LC%. Usually, the LC% of other nano delivery systems is about 1~10%, thus, in our study the LC% -3.3% is reasonable.

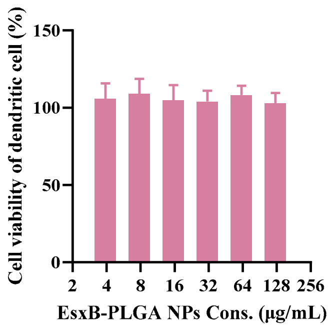

Fig.S5. Biocompatibility evaluation of FITC-EsxB-PLGA NPs in vitro using BMDCs. The data are presented as means ± SD (n = 6, analyzed by one-way ANOVA.). P value < 0.05 was considered as statistically significant (* $p < 0.05$;

** p < 0.01; *** p < 0.001; **** p < 0.0001). The feeding concentration of FITC-EsxB-PLGA NPs was calculated by nanoparticles' concentrations.

The results of Cells viability assays indicated that EsxB-PLGA NPs have good biocompatibility in the concentration range of 4-128 μg/mL.

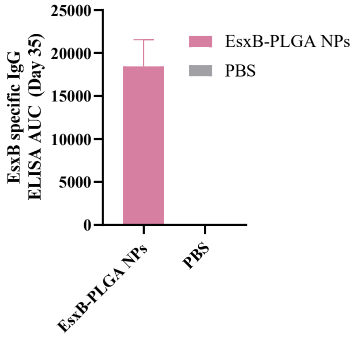

Fig.S6. Serum EsxB-specific IgG titre after subcutaneous injection with EsxB-PLGA NPs/PBS on day 35 (n=6). All data were expressed as mean ± S.D.

We evaluated the effects of NP vaccines on humoral responses in serum 35 days after the final S.C. immunization, and the EsxB-PLGA NPs group exhibited higher EsxB-specific IgG titers compared to PBS groups, the results proved that the mice had been successful immunized.

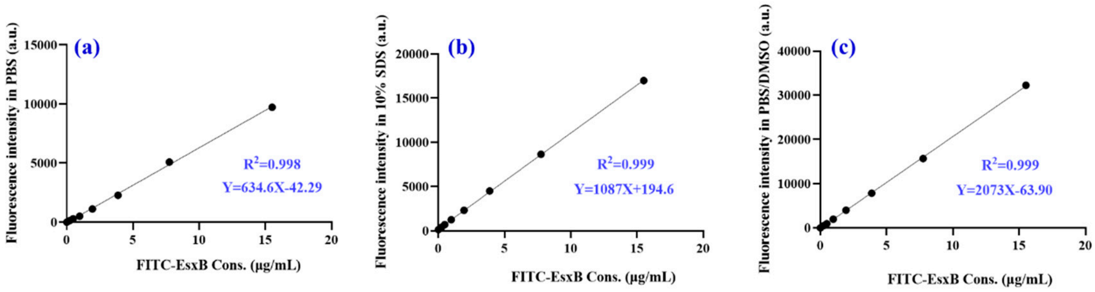

Fig.S7. Standard curve of quantification of FITC-EsxB in different solvents: (a) PBS, (b) 10% SDS, and (c) PBS/DMSO. The plot demonstrates the detection

range of FITC-EsxB from 0 to 15 μg/mL. Fluorescence intensity was measured using a microplate reader with excitation at 490 nm and emission detection at 525 nm (n=3)

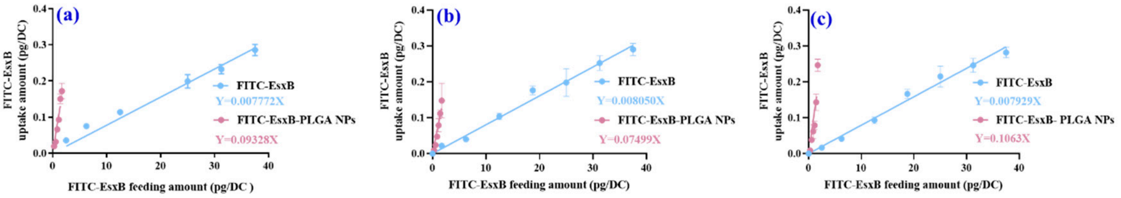

Fig.S8. Quantification of FITC-EsxB or FITC-EsxB-PLGA NPs uptake per cells. (a-c) Triplicate results demonstrate the correlation between antigen feeding amount and antigen uptake amount at the single-cell level. All of the data were reported as the means ± S.D (n=3).

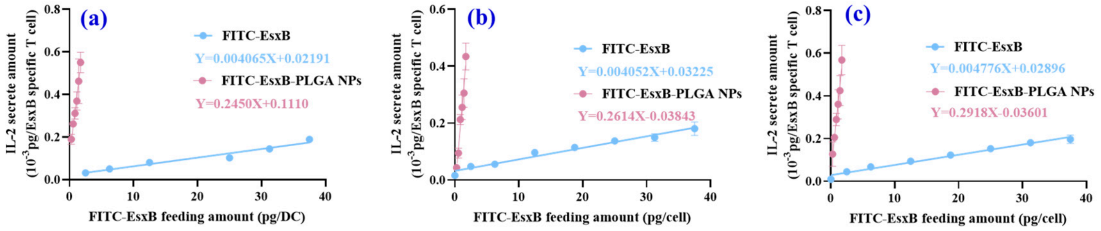

Fig.S9. Quantification of IL-2 secretion as a function of antigen feeding amount per cells. (a-c) Triplicate results demonstrate the correlation between antigen feeding amount per DC and IL-2 secretion per EsxB-specific T cell. All of the data were reported as the means ± S.D (n=3).

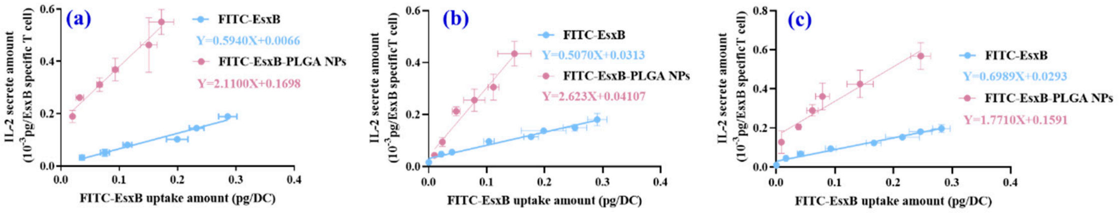

Fig.S10. Quantification of IL-2 secretion as a function of antigen uptake per cells. (a-c) Triplicate results demonstrate the correlation between antigen uptake amount per DC and IL-2 secretion per EsxB-specific T cell. All of the data were reported as the means ± S.D (n=3).

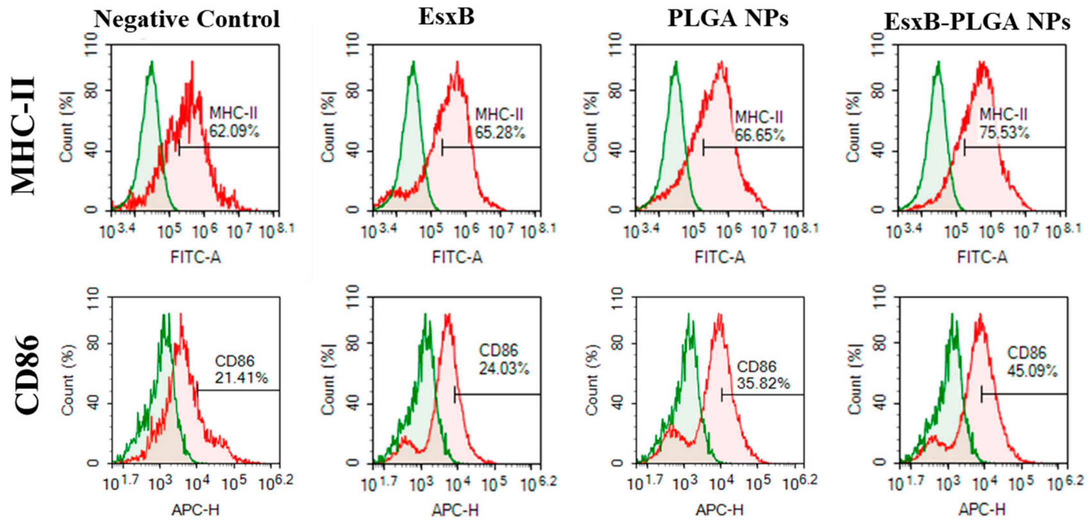

Fig.S11. The representative flow cytometry plots of MHCII/CD86 expressions on BMDCs measured by FCM.

References:

1. Walker, J.M. The bicinchoninic acid (BCA) assay for protein quantitation. Methods in molecular biology (Clifton, N.J.) 1994, 32, 5-8, doi:10.1385/0-89603-268-x:5.
2. Wang, Y.; Qin, B.; Xia, G.; Choi, S.H. FDA's Poly (Lactic-Co-Glycolic Acid) Research Program and Regulatory Outcomes. The AAPS journal 2021, 23, 92, doi:10.1208/s12248-021-00611-y.
3. Chou, P.Y.; Lin, S.Y.; Wu, Y.N.; Shen, C.Y.; Sheu, M.T.; Ho, H.O. Glycosylation of OVA

antigen-loaded PLGA nanoparticles enhances DC-targeting for cancer vaccination. Journal of controlled release : official journal of the Controlled Release Society 2022, 351, 970-988, doi:10.1016/j.jconrel.2022.10.002.
4. Silverstein, R.M.; Bassler, G.C. Spectrometric identification of organic compounds. Journal of Chemical Education 1962, 39, 546, doi:10.1021/ed039p546.
